# GRB2 dimerization mediated by SH2 domain-swapping is critical for T cell signaling and cytokine production

**DOI:** 10.1038/s41598-023-30562-7

**Published:** 2023-03-02

**Authors:** Aline Sandouk, Zhen Xu, Sankar Baruah, Mikaela Tremblay, Jesse B. Hopkins, Srinivas Chakravarthy, Lokesh Gakhar, Nicholas J. Schnicker, Jon C. D. Houtman

**Affiliations:** 1 Interdisciplinary Graduate Program in Immunology, University of Iowa, Iowa City, IA 52242 USA; 2 Protein and Crystallography Facility, University of Iowa, Iowa City, IA 52242 USA; 3 Department of Microbiology and Immunology, University of Iowa, Iowa City, IA 52242 USA; 4 Department of Biochemistry and Molecular Biology, Roy J. and Lucille A. Carver College of Medicine, University of Iowa, Iowa City, IA 52242 USA; 5 Biophysics Collaborative Access Team, Argonne National Laboratory, Argonne, IL 60439 USA

**Keywords:** Signal transduction, Cell signalling, Molecular modelling, SAXS, Intracellular signalling peptides and proteins

## Abstract

GRB2 is an adaptor protein required for facilitating cytoplasmic signaling complexes from a wide array of binding partners. GRB2 has been reported to exist in either a monomeric or dimeric state in crystal and solution. GRB2 dimers are formed by the exchange of protein segments between domains, otherwise known as “domain-swapping”. Swapping has been described between SH2 and C-terminal SH3 domains in the full-length structure of GRB2 (SH2/C–SH3 domain-swapped dimer), as well as between α-helixes in isolated GRB2 SH2 domains (SH2/SH2 domain-swapped dimer). Interestingly, SH2/SH2 domain-swapping has not been observed within the full-length protein, nor have the functional influences of this novel oligomeric conformation been explored. We herein generated a model of full-length GRB2 dimer with an SH2/SH2 domain-swapped conformation supported by in-line SEC–MALS–SAXS analyses. This conformation is consistent with the previously reported truncated GRB2 SH2/SH2 domain-swapped dimer but different from the previously reported, full-length SH2/C-terminal SH3 (C–SH3) domain-swapped dimer. Our model is also validated by several novel full-length GRB2 mutants that favor either a monomeric or a dimeric state through mutations within the SH2 domain that abrogate or promote SH2/SH2 domain-swapping. GRB2 knockdown and re-expression of selected monomeric and dimeric mutants in a T cell lymphoma cell line led to notable defects in clustering of the adaptor protein LAT and IL-2 release in response to TCR stimulation. These results mirrored similarly-impaired IL-2 release in GRB2-deficient cells. These studies show that a novel dimeric GRB2 conformation with domain-swapping between SH2 domains and monomer/dimer transitions are critical for GRB2 to facilitate early signaling complexes in human T cells.

## Introduction

**Figure 1:**
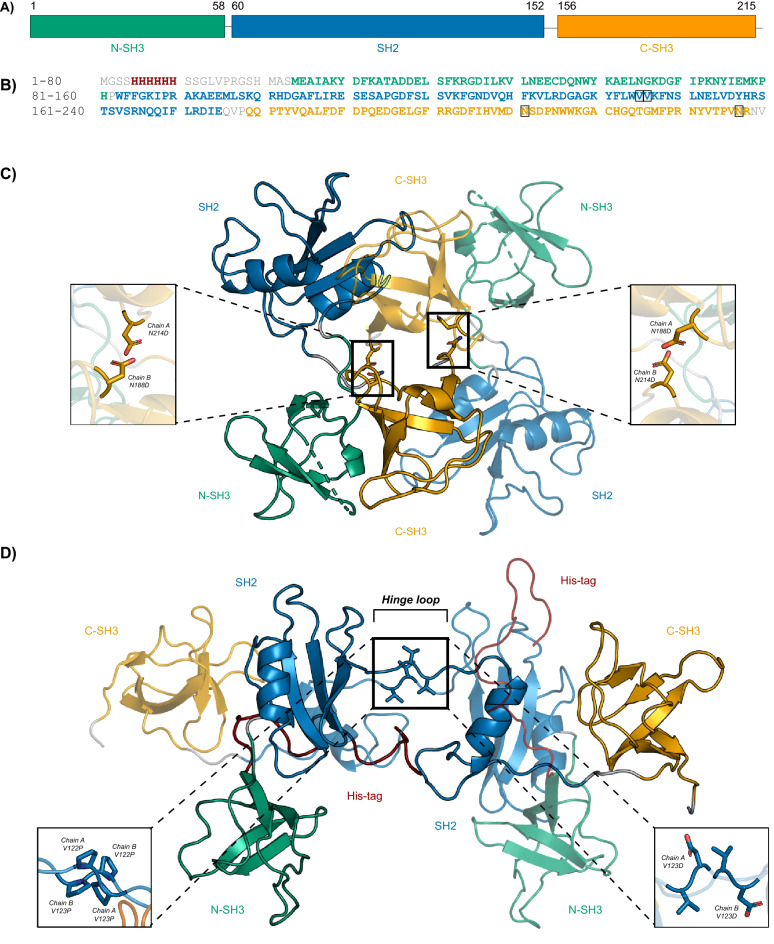
Structural models of full-length human wild-type GRB2. (**A**) A schematic is shown depicting the domain organization of full-length GRB2 with corresponding amino acid numbering. N-terminal SH3, central SH2, and C-terminal SH3 domains are shown in green, blue, and orange, respectively. (**B**) The amino acid sequence of recombinant His-tagged GRB2 is shown with segments colored as in the schematic shown in Panel A with linkers in grey and poly-histidine-tag in red. Residues targeted for mutation are boxed in black. (**C**) Ribbon representation of a GRB2 dimer crystal structure (PDB: 1GRI) with SH2/C-SH3-mediated domain-swapping. One protomer is depicted partially transparent for contrast. Inset shows conversion of native N188 and N214 sites to Asp residues as mutations reported to abolish dimerization. (**D**) Ribbon representation of the proposed SH2/SH2-mediated domain-swapped dimer model with C-terminal α-helix segment exchanged developed from a combination of an SH2/SH2 domain-swapped dimer consisting of the isolated SH2 domain (PDB: 6ICH) supplemented with the N- and C–SH3 domains derived from the full length GRB2 crystal structure (PDB: 1GRI). Insets show the V122P/V123P mutation to induce dimerization (left) and the V123D mutation to abolish dimerization (right). Images were generated by the authors using PyMOL.

Growth factor receptor-bound protein 2 (GRB2) is an ubiquitously-expressed, non-catalytic adaptor protein^1^. Monomeric GRB2 is 25 kDa and consists of a central Src Homology 2 (SH2) (residues 60–152) domain flanked by two Src Homology 3 (SH3) domains (residues 1–58 and 156–215, respectively) (Fig. 1A,B)^2^. The most well-studied role of GRB2 is its ability to bring disparate signaling molecules into close proximity and facilitate the formation of complexes important for signal transduction^2^. The SH2 domain binds specific phosphorylated tyrosine residues on proteins, allowing GRB2 to associate broadly with molecules at the cell surface and plasma membrane, including growth factor receptors, cytokine receptors, CD28 co-stimulatory receptor, T cell receptor (TCR) ζ chains, and Linker for activation of T cells (LAT), among others^2–16^. The terminal SH3 domains bind proline-rich regions on downstream signaling molecules such as SOS1, c-Cbl, Vav, and SLP-76, and more^10,12,15,17–30^. Engagement of the TCR leads to phosphorylation of multiple tyrosine residues on the adaptor protein LAT, which in turn, serve as docking sites for various SH2 domain-bearing binding partners, including GRB2^31–35^. Proper signal transmission depends critically on these early phosphorylation and recruitment events; as such, disruption of these events may compromise downstream cellular output. Our lab has described a novel role for GRB2 in stabilizing large, multimeric signaling complexes at the plasma membrane of human CD4+ T cells^8^. Specifically, this function is mediated by the interaction between SOS1 with two GRB2 molecules and the binding of GRB2 to three possible sites on LAT. These interactions trigger LAT oligomerization into micro-clusters and facilitates the recruitment of additional signaling molecules^8,36^. The broad range of GRB2 binding partners makes it indispensable for induction of essential cellular processes ranging from metabolism to proliferation and differentiation^9,37–39^. Accordingly, aberrant GRB2 function and the resulting disruptions of GRB2-mediated signaling, in particular the Ras/MAPK pathway, have been implicated in malignant transformation across cell types and organ systems^40–46^. The specific importance of GRB2’s conformation and modular SH3–SH2–SH3 structure has been shown in multiple studies where modifications of GRB2 ranging from point mutations to full domain deletions lead to interesting changes in overall protein topology and function, and consequently, changes in downstream cellular output^47–56^. For these reasons, the structure and function of GRB2 and any modulation thereof have become major areas of investigation for novel interventions against human disease.

GRB2 structure has been intensively characterized, both in isolation and in association with cognate ligands (Supplemental Table 1)^57–72^. Surprisingly, only one full-length structure of GRB2 has ever been reported, which was determined by X-ray crystallography (XRC) at a resolution of 3.1 Å shortly after the protein was originally discovered^71^. In the crystal structure (PDB: 1GRI), GRB2 is in an unbound and globular dimeric form with an anti-parallel protomer arrangement and significant inter-molecular contacts between one protomer’s SH2 domain and the C–SH3 domain of the other protomer (Fig. 1C)^70,71^. Furthermore, each protomer is also a compact structure in which the individual domains are engaged in several intra-molecular interactions. Based on the SH2/C–SH3 domain-swapped dimer crystal structure, one study reported on a construct consisting of Asn-to-Asp mutations at positions 188 and 214 purported to destabilize the SH2/C–SH3 domain-swapped dimer by introducing charge at the dimer interface (Fig. 1C)^73^. The authors reported that the N188D/N214D GRB2 monomer was capable of binding SOS1 and in doing so, upregulating MAPK signaling, whereas GRB2 dimer was not^73^. However, the N188D/N214D construct was not extensively characterized using in vitro biophysical analyses. Beyond the XRC study, there are very few other structural studies involving full-length GRB2. One such study analyzed the full-length protein by Nuclear Magnetic Resonance (NMR) and Small-Angle X-ray Scattering (SAXS)^72^. In contrast to the SH2/C–SH3 domain-swapped dimer crystal structure, the authors report that GRB2 in solution with its individual domains complexed with their respective ligands is in a flexible and extended monomeric form with no detectable inter-domain contacts. They also report that the domains within the full-length protein are structurally similar to their isolated domain counterparts, further suggesting that the domains do not influence each other’s conformations. The authors conclude that ligand-bound GRB2 in solution exists as a collection of conformations that is not adequately represented by the static molecule depicted by the crystal structure (PDB: 1GRI). Together, these studies indicate that the current record reflects neither the complete structural and conformational picture of full-length GRB2 nor the cellular purpose of GRB2 oligomerization.

Although the body of work on full-length GRB2 is lacking, several other studies have investigated the structures of the isolated domains with and without associated ligands (Supplemental Table 1). Interestingly, a number of studies on the isolated SH2 domain have reported a dimer formed by inter-SH2 domain-swapping that has so far not been reported in the full-length protein^57,58,60,61,66–69^. The basis for the SH2/SH2 domain-swapped dimer is the extension of a C-terminal α-helix and part of the preceding hinge loop (Trp121–Val123) connecting the C-terminal α-helix to the rest of the SH2 domain toward an adjacent SH2 protomer, such that one SH2 domain swaps its α-helix for that of another SH2 domain. Intriguingly, this form of inter-SH2 dimerization is accompanied by either increases or decreases in SH2 binding affinity for phospho-peptides in comparison with the isolated SH2 domain. Specifically, the SH2/SH2 domain-swapped dimer was found to bind an Shc-derived ligand with a lower affinity and a CD28-derived ligand with a higher affinity when compared to the SH2 monomer^60,69^. Of note, the un-swapped segments of the SH2 domains in the SH2/SH2 domain-swapped dimer are virtually identical in structure to the isolated domain, with the exception of the hinge loop that is N-terminal to the swapped α-helix, suggesting that the loop, rather than overall domain topology of the protein, governs the interconversion of SH2 dimer and monomer. Importantly, the hinge loop mediates the SH2 domain’s ligand-binding specificity, specifically Trp121^58,59,74–76^. Though important for engaging ligands, Trp121 has been shown to be irrelevant for dimerization, pointing to a potential role for the remaining residues of the hinge loop, Val122 and Val123, in mediating domain-swapping and dimerization^60,68^. However, no studies have ever been undertaken to determine their contribution to domain-swapping, nor has SH2/SH2 domain-swapping been explored as a form of full-length GRB2 dimerization.

Given the paucity of reporting on full-length GRB2 and the reported structural and functional peculiarities of the SH2/SH2 domain-swapped dimer, we set out to better characterize GRB2’s conformational potential. We first examined the structure of human full-length, ligand-free GRB2 in solution and determined whether SH2/SH2 domain-swapping occurs in the full-length protein. We then investigated the structure of SH2/SH2 domain-swapping in the full-length protein, and in particular, the role of Val122 and Val123 in SH2/SH2 domain-swapping. We developed GRB2 constructs with mutations at the hinge loop intended to stabilize either a closed or extended loop orientation and consequently, abolish or induce SH2/SH2 domain-swapping. We also replicated the previously-reported N188/N214 construct as a predominantly monomeric protein. From the existing literature, we generated a model of full-length GRB2 dimer with SH2/SH2 domain-swapping and used biophysical analyses to verify it against the GRB2 crystal structure (PDB: 1GRI), the N188/N214 mutant, and the V122/V123 hinge loop mutants. To complement our in vitro and in silico work, we identified effects of the hinge loop mutants on T cell function. As a result of this work, we report a model of GRB2 dimer with SH2/SH2 domain-swapping as an underlying mechanism for dimerization, which, when disrupted, significantly alters signal transduction and a impairs T cell cytokine output.

## Results

In our investigation of the remaining hinge loop residues, we designed constructs intended to stabilize either a closed or extended loop orientation and consequently, abolish or induce SH2/SH2 domain-swapping. Given that Pro residues often occur in hinge loops and are important in the swapping process, we mutated Val122 and Val123 to Pro residues in order to generate a stable dimeric GRB2 construct (Fig. 1D)^77–85^. We hypothesized that the strain introduced into the loop by the Pro side chains would induce opening of the loop and lower the energetic barrier to SH2/SH2 domain-swapping. Conversely, to generate a stable monomeric construct, we mutated Val123 to an Asp residue. We hypothesized that the introduction of a charged residue at the SH2/SH2 domain-swapped dimer interface would destabilize the domain-swapped dimer and favor the lower-energy monomer. In addition to the V123D and V122P/V123P constructs, we replicated the previously-reported N188D/N214D construct as a stable monomer control for our studies.

All mutants were expressed as poly-histidine-tagged (His-tagged) proteins in *E. coli* and isolated the constructs from bacterial lysates by affinity (Ni–NTA) chromatography. Except for V122P/V123P, all other samples eluted as a single peak from Ni–NTA columns. SDS-PAGE analyses of the eluates showed homogeneous protein of ~ 27 kDa with virtually no impurities, whereas the V122P/V123P mutant also showed several distinct products of < 27 kDa (Supplemental Fig. 1 ).The smaller products of the V122P/V123P sample may represent failed attempts at bacterial translation, the products of which were nonetheless captured by the Ni–NTA column along with full-length protein since the construct was His-tagged at the N-terminus. Otherwise, the molecular weight (MW) of 27 kDa is consistent with the expected value for GRB2 monomer as calculated from the wild-type (WT) amino acid sequence together with N-terminal His-tag, thrombin cleavage site, and intervening residues (Fig. 1B).

**Figure 2:**
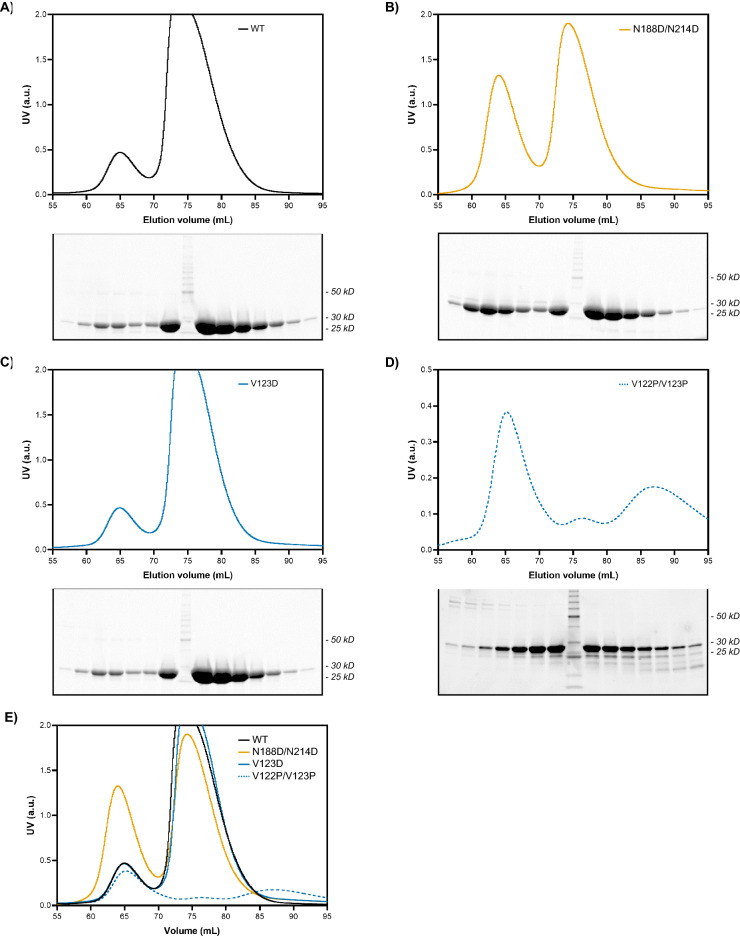
SEC and SDS-PAGE analysis of GRB2 WT and mutants. The SEC profile and SDS PAGE analysis of fractions spanning chromatographic peaks are shown for (**A**) GRB2 WT, (**B**) N188D/N214D, (**C**) V123D putative monomer, and (**D**) V122P/V123P putative dimer. For V122P/V123P, scale is adjusted to enhance peak prominence. (**E**) An overlay of all construct SEC profiles is shown.

Further resolution of the samples by size-exclusion chromatography (SEC) gave two separate and relatively well-separated peaks with no evidence of aggregation or degradation for all mutants except for V122P/V123P (Fig. 2; Supplemental Fig. 2). GRB2 monomer dominates the WT population, on the order of ~ 20% dimer and ~ 80% monomer, suggesting that the dimer is a less favorable state than the monomer (Fig. 2A). A similar monomer–dimer distribution was observed for the V123D mutant as for the WT with an unexpected dimer peak, although it was highly unstable and precipitated readily (Fig. 2C). Contrary to what was expected to be a single monomer peak, the N188D/N214D mutant generated a prominent dimer peak, on the order of ~ 40% dimer and ~ 60% monomer (Fig. 2B). The substantial dimer population may represent other dimeric conformations of GRB2 that are not abolished by the N188D/N214D mutations and are otherwise masked by the predominant SH2/C-SH3 domain-swapped dimer. The V122P/V123P mutant generated a predominantly dimeric population on isolation, with ~ 80% dimer and ~ 20% monomer (Fig. 2D). As noted previously, the additional peaks likely represent failed attempts at translating the V122P/V123P construct that would have been otherwise excluded from isolation on the Ni–NTA column if not for the N-terminal His-tag. An overlay of the SEC profiles normalized to the monomer peak highlights the considerable differences in dimer-to-monomer ratios of the different constructs (Fig. 2E). SDS–PAGE analyses of the eluates showed bands of high purity at ~ 27 kDa across both peaks for WT, N188D/N214D, and V123D (Fig. 2A, 2B, and 2C). For the V122P/V123P mutant, strong bands were also observed at ~ 27 kD, though accompanied by bands greater than and less than 27 kDa (Fig. 2D).

**Figure 3:**
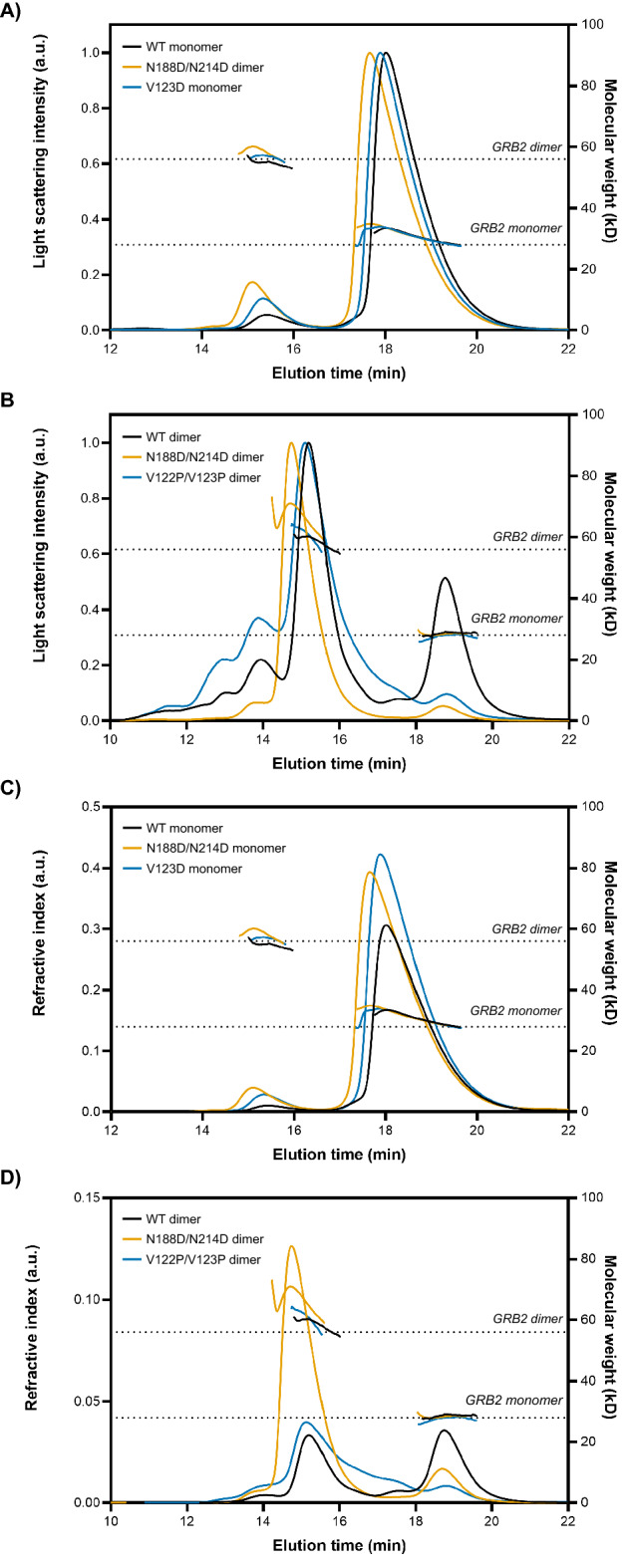
SEC-MALS analysis of GRB2 mutants. Overlays of GRB2 WT, N188D/N214D, and V123D monomers are shown in black, orange, and blue, respectively, with (**A**) light scattering or (**C**) refractive index plotted against elution time. Overlays of GRB2 WT, N188D/N214D, and V122P/V123P dimers are shown in black, orange, and blue, respectively, with (**B**) light scattering or (**D**) refractive index plotted against elution time. Dashed lines denote the expected molecular weights of GRB2 monomer (~ 28 kDa) and dimer (~ 56 kDa).

**Figure 4:**
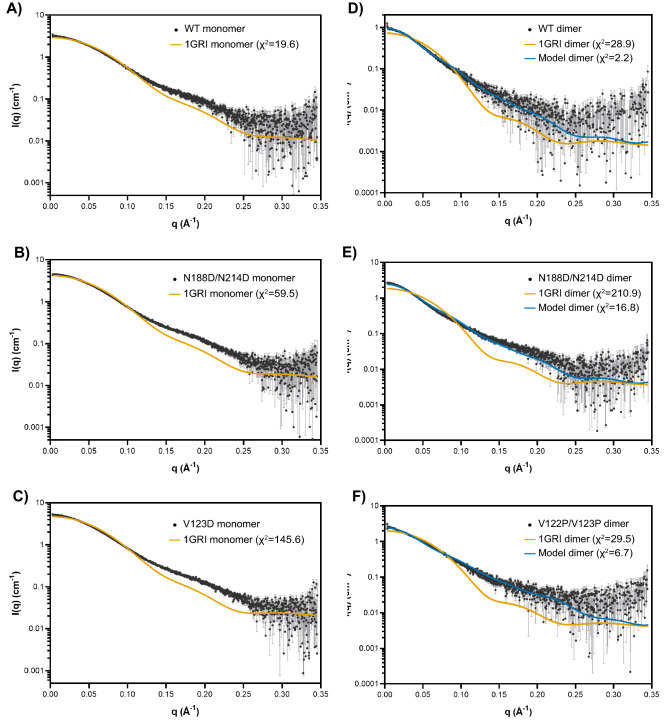
CRYSOL fitting of crystal structure (PDB: 1GRI) to experimental SAXS scattering profiles. SAXS profiles of the purified monomer and dimer from GRB2 WT and mutants are superimposed with theoretical profiles generated by CRYSOL of either monomer or dimer (orange) from the crystal structure (PDB: 1GRI) or the proposed SH2/SH2-mediated domain-swapped dimer model (blue). (**A**) WT monomer versus 1GRI monomer; (**B**) N188D/N214D monomer versus 1GRI monomer; (**C**) V123D putative monomer versus 1GRI monomer; (**D**) WT dimer versus 1GRI (SH2/C-SH3) domain-swapped dimer or SH2/SH2 domain-swapped dimer; (E) N188D/N214D dimer versus 1GRI dimer or SH2/SH2 domain-swapped dimer; and (**F**) V122P/V123P putative dimer versus 1GRI dimer or SH2/SH2 domain-swapped dimer.

The structure and oligomeric states of the WT and mutants of full-length GRB2 were further characterized by SEC in-line with multi-angle light scattering (MALS) and SAXS. All isolated monomer and dimer subpopulations exhibited some degree of re-equilibration of monomer and dimer (Fig. 3; Supplemental Fig. 3). Meanwhile, the experimental MWs of WT, N188D/N214D, and V123D monomers and dimers were determined by MALS. The experimental MWs were consistent with the theoretical MWs of monomeric and dimeric GRB2 at 27.6 kDa and 55.3 kDa, respectively (Table 1). Using SAXS, we observed no confounding inter-particle effects for all samples except V122P/V123P, as Guinier regions at low *q* range showed good linearity that indicates good quality data. Conversely, analysis of the V122P/V123P sample indicated some aggregation (Supplemental Fig. 4). SAXS profiles of WT and mutant samples were compared against the theoretical monomer or dimer profile computed from the GRB2 crystal structure (PDB: 1GRI) using the program CRYSOL^86,87^. A comparison of monomer from 1GRI to the WT, N188D/N214D, and V123D monomers yielded χ^2^ values of 19.6, 59.5, and 145.6, respectively (Fig. 4A,B,C). These values suggest poor fitting of the crystal structure to the conformation of the proteins in solution. Similarly, a comparison of the 1GRI dimer to the WT, N188D/N214D, and V122P/V123P dimers yielded χ^2^ values of 28.9, 210.9, and 29.5, respectively, again indicating poor fitting between the crystal structure and the conformation of the proteins in solution (Fig. 4D,E,F). These results suggest that neither the monomer nor the dimer depicted by the crystal structure of GRB2 accurately represents the conformation of GRB2 in solution.

**Table 1:** Summary of SEC–MALS–SAXS analysis of GRB2 WT and mutants.

Sample	Conc.* (mg/mL)	Conc.* (uM)	MW_Exp_ (kDa)	MW_MALS_ (kDa)	MW_SAXS_ (kDa)	*R*_g_** (Å)	*R*_g_*** (Å)	*D*_max_ (Å)	Corrected porod volume (Å^3^)
WT monomer	8.28	298.9	27.7	30.8 ± 1.8	28.6	24.9 ± 0.1	25.4	90	34,500
WT dimer	4.46	80.7	55.3	59.3 ± 3.3	74.6	36.9 ± 0.1	38.4	140	89,900
N188D/N214D monomer	8.37	298.6	27.7	31.9 ± 2.1	30.1	25.2 ± 0.1	25.7	95	36,200
N188D/N214D dimer	2.84	51.4	55.3	64.8 ± 4.7	88.1	40.9 ± 0.1	42.3	150	105,000
V123D monomer	8.68	313.4	27.7	30.8 ± 2.2	31.8	26.1 ± 0.1	27.0	100	38,400
V122P/V123P dimer	4.00	72.3	55.3	60.7 ± 2.7	57.9	36.7 ± 0.1	47.9	204	69,800

Conc.—sample concentration at the time of SEC injection; MW_Exp_—expected molecular weight calculated from primary amino acid sequence; MW_MALS_—molecular weight determined by MALS; MW_SAXS_—molecular weight estimated from SAXS using Porod volume; *R*_g_**—radius of gyration estimated in reciprocal space from the Guinier plot; *R*_g_***—radius of gyration estimated in real space using the program GNOM; *D*_max_—maximum dimension estimated using the program GNOM; Corrected Porod volume—particle volume calculated from Porod’s law.

The incompatibility of the crystal structure and the solution proteins led us to ask whether a GRB2 dimer with SH2/SH2 domain-swapping might better fit our SAXS data. We generated a model of SH2/SH2 domain-swapped full-length GRB2 dimer by combining an SH2/SH2 domain-swapped dimer consisting of the isolated SH2 domain (PDB: 6ICH) and the N- and C–SH3 domains derived from the full-length GRB2 crystal structure (PDB: 1GRI) (Fig. 1D)^60,70,71^. In contrast to the crystal structure, fitting of the full-length SH2/SH2 domain-swapped GRB2 dimer to the WT, N188D/N214D, and V122P/V123P dimers gave χ^2^ values of 2.2, 16.8, and 6.7, indicating vastly improved fits (Fig. 4D,E,F). Taken together, these findings raised questions regarding the crystal structure of GRB2 and the overall conformational states of the protein in solution.

**Figure 5:**
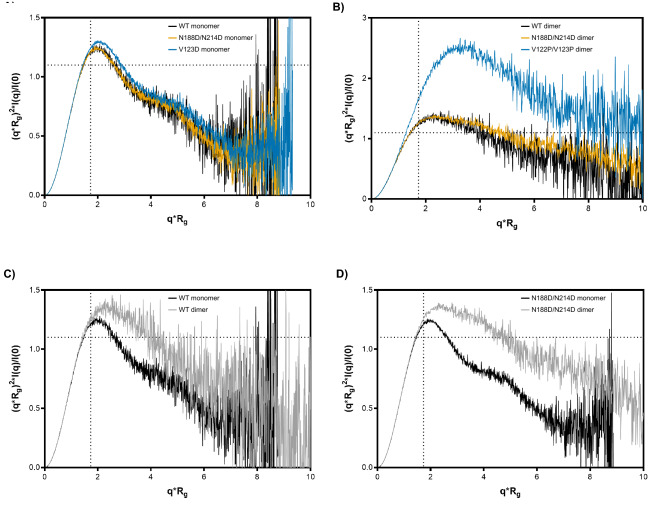
Kratky analysis for flexibility and compactness. Overlays of normalized Krakty plots are shown for monomers (**A**) and dimers (**B**). Comparisons between WT monomer and dimer (**C**) and N188D/N214D monomer and dimer (**D**) are shown. Dotted lines indicate where the maxima are expected to occur for an ideal, compact, globular protein (qR_g_ ~ 1.73 for the peak position; ~ 1.1 for the peak height).

For a qualitative assessment of each protein’s globularity, we examined the scattering intensities in mid- and high-q regions through Kratky analysis. The height of the mid-q peak (q∼0.1 Å^−1^) and the slope of the higher q scattering intensities (q > 0.1 Å^−1^) of a Kratky plot provides insight into the degree of compactness and unfolding of a protein. A well-folded, globular structure is characterized by a symmetric peak in the mid-q region followed by a negatively-sloped tail converging on the x-axis. In contrast, an unfolded, disordered, or extended structure exhibits a peak maximum that continues to rise and plateaus at high q. In between, the curves of partially folded proteins show both folded and unfolded characteristics; for example, a peak maximum followed by longer, shallower, more linear tail suggests a more unfolded structure. All normalized, dimensionless Kratky analyses are shown in Fig. 5. The Kratky plots for the WT, N188D/N214D, and V123D monomers show the first half of a bell-shaped curve with a well-defined maximum, followed a second, smaller peak (Fig. 5A). This multi-peak profile is consistent with a multi-domain structure with globular domains connected by flexible linkers. The high similarity between the monomer curves suggests that despite the introduced mutations, the monomers are of similar structure. In contrast, the Kratky plots for the WT, N188D/N214D, and V122P/V123P dimers also exhibit well-defined maxima (Fig. 5B). However, unlike the monomers, the WT, N188D/N214D, and V122P/V123P maxima are followed by longer, flatter, less strongly-sloped curves. These profiles are instead suggestive of structures that are less compact and partially unfolded. Notably, the peak height of the V122P/V123P maximum is markedly increased compared to those of its WT and N188D/N214D dimers; however, while this effect may be due to increased flexibility of the V122P/V123P mutant, it may also be induced by the presence of sample aggregates, as first seen in Fig. 2D. However, these WT and N188D/N214D dimer plots generally indicate that the dimers are more unfolded, more flexible structures relative to their monomer in counterparts. This difference is more readily apparent when comparing the WT and N188D/N214D dimers to their respective monomers (Fig. 5C,D). Taken together, these findings indicate a high degree of compactness and globularity among the monomer samples, in contrast to the dimer samples, which exhibit more flexibility.

**Figure 6:**
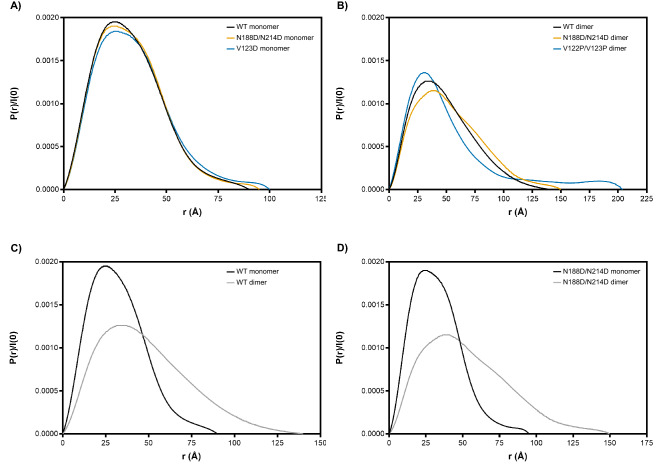
P(r) function analysis for maximum dimension. Overlays of P(r) function plots are shown for monomers (**A**) and dimers (**B**). Comparisons between WT monomer and dimer (**C**) and N188D/N214D monomer and dimer (**D**) are also shown.

SAXS data were also used to calculate the pair-wise distance distribution function, or *P(r)* function, by taking an indirect Fourier transform (IFT) of the scattering curve using GNOM from the ATSAS data analysis program^86^. The *P(r)* function is represented as a histogram of all inter-electron distances within the particle and is used to estimate the maximum linear dimension of the particle, *D*_*max*_. This parameter is defined as where the *P(r)* curve decays smoothly to zero without significant oscillations in the curve. Globular proteins exhibit a single peak that is bell-shaped. Furthermore, the more spherical the particle, the more symmetric the *P(r)* function will be. Otherwise, non-spherical, elongated, or irregular proteins have an extended curve with a longer tail and may exhibit multiple peaks if modular. All of the dimeric samples were significantly more extended in comparison with their monomeric counterparts. The *D*_*max*_ values of the WT dimer and monomer were 140 and 90 Å, respectively (Fig. 6C and Table 1). Similarly, the N188D/N214D dimer and monomer showed *D*_*max*_ values of 150 and 95 Å, respectively (Fig. 6D and Table 1). Lastly, the V122P/V123P dimer exhibited a *D*_*max*_ of 204 Å and the V123D monomer a *D*_*max*_ 100 Å (Table 1). Relative to each other, we observed that the WT monomer is more compact than the N188D/N214D monomer, which in turn is more compact than the V123D monomer (Fig. 6A). However, we interpreted these discrepancies to be subtle and the monomers to be of overall similar compactness. With respect to the dimers, we observed a similar pattern with the WT being more compact than the N188D/N214D mutant, which was more compact than the V122P/V123P dimer (Fig. 6B). While this hints at the possibility that the V122P/V123P mutant is the most extended of the mutants, a markedly increased *D*_*max*_ may also be characteristic of aggregation; thus, here too, we cannot confidently conclude that the V122P/V123P mutant is more flexible than its WT and N188D/N214D counterparts. The shape of the *P(r)* function for the WT dimer in comparison to the WT monomer suggests that the dimer is far more extended and flexible than the monomer (Fig. 6C). This finding was reinforced by the increased *D*_*max*_ of the dimer (140 Å) in comparison with that of the monomer (90 Å) (Table 1). We saw a similar pattern in *P(r)* function and *D*_*max*_ values comparing the N188D/N214D dimer (150 Å) with the N188D/N214D monomer (90 Å) (Fig. 6D and Table 1). Taken together, these findings indicate subtle but appreciable differences in the overall dimensions of the different mutants and support earlier observations that dimeric conformations of GRB2 are overall more extended and flexible than the monomeric conformations.

Guinier analysis and the *P(r)* function may both be used to assess the overall size of the protein through estimation of the radius of gyration, *R*_*g*_. *R*_*g*_ generally serves a measure of the way in which a protein’s mass is distributed about the protein’s center-of-mass. Importantly, *R*_*g*_ calculated from the *P(r)* function has the advantage of being derived from all available data points on the scattering curve, rather than the low-q region as in the Guinier approximation^88^. Agreement between the Guinier-derived *R*_*g*_ and *P(r)* function-derived *R*_*g*_ values reinforced that good quality data were collected (Table 1). One exception was V122P/V123P, which gave a *P(r)* function *R*_*g*_ that was larger than the Guinier *R*_*g*_. As mentioned previously, this difference may be due to increased flexibility as well as aggregation. Overall, these findings indicate minor differences when comparing the monomeric counterparts to each other and the dimeric counterparts to each other, but support earlier observations that dimeric conformations of GRB2 are overall more extended than monomeric conformations.

**Figure 7:**
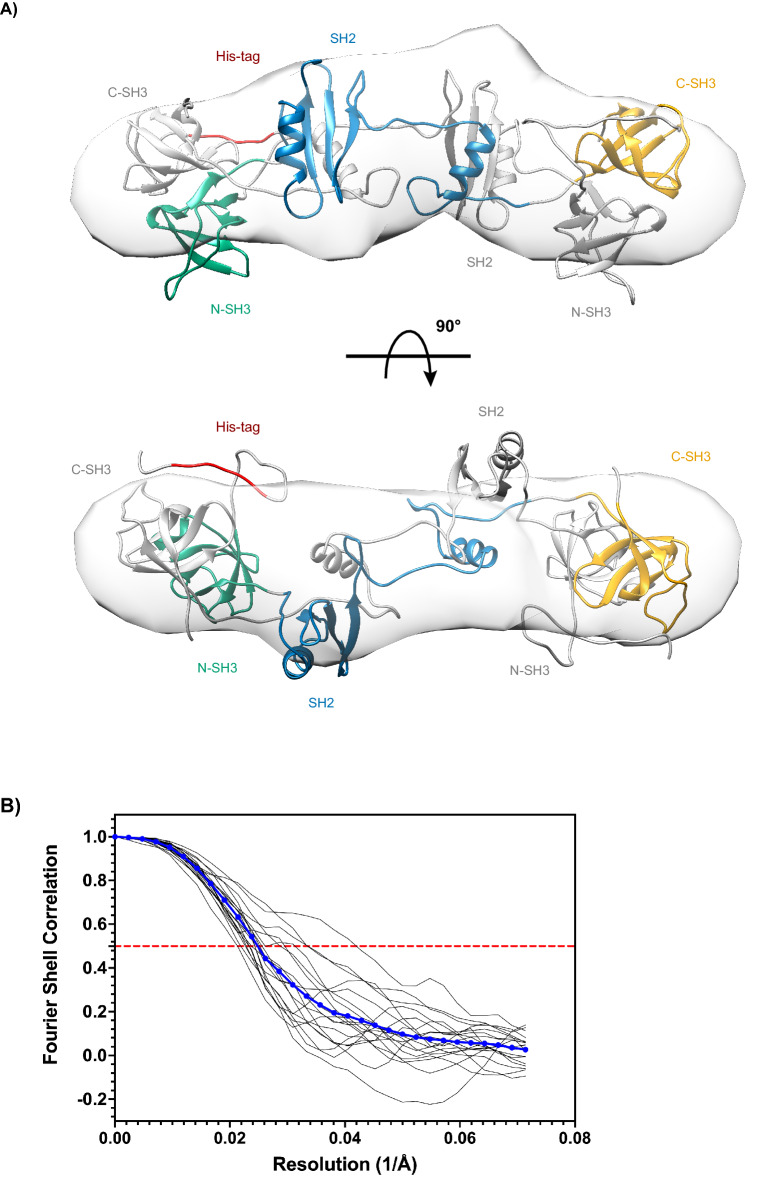
Electron density reconstruction of WT GRB2 dimer in solution calculated from scattering data using DENSS. (**A**) Orthogonal views with 90° rotation of the proposed full-length SH2/SH2-mediated domain-swapped dimer model superposed onto the electron density reconstruction are shown. One protomer is depicted as in Fig. 1, with the N-terminal SH3 domain in green, central SH2 domain in blue, and C-terminal SH3 domain in orange, linkers in grey, and poly-histidine-tag in red. The other protomer is shown entirely in grey for contrast. (**B**) The averaged FSC curve as a function of reciprocal electron density resolution is shown. The red, dotted line indicates the resolution limit, where the FSC falls below 0.5.

To further visualize the structural organization of the WT dimer in solution, we calculated the electron density from the solution scattering data using the program DENSS, which is embedded in the BioXTAS RAW software^89^. A total of 20 independent iterations were averaged to generate a consensus reconstruction. The resulting three-dimensional envelope exhibited a lobular, elongated shape with dimensions grossly similar to our proposed SH2/SH2 domain-swapper dimer model. When directly superposed onto our model, we observed the reconstruction and the model to be consistent, with each of the model’s domains mostly situated inside each of the three lobes of the extended envelope (Fig. 7A). The reconstruction is in good agreement with the *P(r)* function, which also indicated an elongated structure (Fig. 6). Furthermore, the quality of the DENSS reconstruction was assessed by the standard Fourier shell correlation (FSC) approach. The averaged FSC resolution curve obtained was satisfactory, remaining one at low frequencies, followed by a semi-Gaussian fall-off, dropping to zero at ~ 2/3 of the maximum frequency, and oscillating around zero at high frequencies (Fig. 7B). Together with our other findings, the ab initio modeling of the solution scattering data further supports our SH2/SH2 domain-swapped dimer model, which is distinct from the crystal structure (PDB 1GRI).

**Figure 8:**
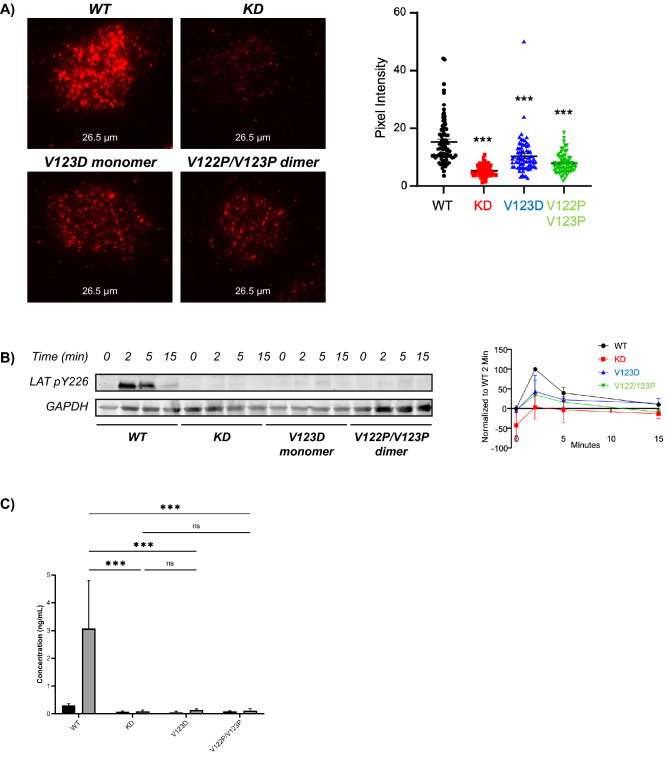
Effects of monomer/dimer disruption on cellular function in GRB2-deficient (knockdown, or KD) and mutant GRB2-expressing T cells. (**A**) Phosphorylated LAT clustering at the plasma membrane was analyzed by TIRF microscopy (N = 3 with 50 + analyzed cells) and quantified. (**B**) LAT phosphorylation over time was analyzed in response to stimulation with 1 μg/mL anti-CD3 antibody, using Western blot and densitometric quantification (N = 2–3). (**C**) Secretion of IL-2 in response to stimulation with 1 μg/mL anti-CD3 antibody for 24 h was analyzed by ELISA (N = 3). Two-way ANOVA analysis was used to determine significance, denoted by ** as *P* < 0.01 and *** as *P* < 0.001.

Currently, there is no evidence that full-length GRB2 forms an SH2/SH2 domain-swapped dimer in vivo. We sought to determine whether the V123D and V122P/V123P mutations had physiological relevance and/or pathological consequences. We used lentiviruses to express the mutants in HUT 78 T lymphocytes that were deficient in GRB2. We found that the WT, V123D, and V122P/V123P mutants expressed similar levels of GRB2, while GRB2-deficient T cells had markedly reduced GRB2 levels (Supplemental Fig. 5). We found that the clustering of phosphorylated LAT at the plasma membrane is reduced in cells expressing either of the V123D and V122P/V123P GRB2 mutants, albeit to a lesser degree than the effect in GRB2-deficient cells, compared to WT GRB2-expressing cells (Fig. 8A). We also found that bulk LAT phosphorylation was reduced in GRB2-deficient cells and cells expressing the V123D and V122P/V123P GRB2 mutants compared to WT GRB2-expressing cells (Fig. 8B; Supplemental Fig. 6). Most critically, we observed a near-total loss of IL-2 production in GRB2-deficient and mutant-expressing cells compared to WT GRB2-expressing cells (Fig. 8C). These data show that TCR-induced LAT clustering and cytokine production is dependent on the dynamic equilibrium between the monomeric and dimeric forms of GRB2.

## Discussion

GRB2 is a critical actor in normal cell function, as well as in pathological processes leading to various cancers and chronic diseases. However, the current body of knowledge of GRB2 structure as it relates to the protein’s function remains incomplete, in particular the role of SH2 domain-swapping form of GRB2 dimerization. In our study, we undertook experiments to better understand the structure of GRB2 and to identify their effects in a cellular context. In our studies, we found GRB2 to be well-resolved into two separate species determined reliably to be a monomer and a dimer. We sought to influence this distribution by targeting a known but poorly-studied hinge region for mutations with the intention of abolishing or inducing dimerization. Importantly, we found that the C–SH3/SH2 domain-swapped crystal structure is incompatible with our SAXS data, neither of WT proteins nor the mutants. We observed a high degree of flexibility among all constructs, but distinct patterns of globularity among monomers and of extendedness among dimers. This contrasted sharply with the crystal structure, which implies that GRB2 dimer is inflexible and compact. While this does not entirely exclude the C–SH3/SH2 domain-swapped dimer from the spectrum of GRB2 structures, it does suggest that the crystal structure does not represent the protein’s primary conformation in solution. We speculate that the discrepancies between the crystal structure and the solution proteins may be the result of the process of crystallization, which exerts a selective pressure on proteins such that those conformational states that are amenable to close packing in a crystal lattice predominate over other states of the proteins. Inconsistencies between crystal structures and solution structures have been previously attributed to crystal packing forces^90–94^. However, the possibility that the SH2/C–SH3 domain-swapped dimer represents a species that was disproportionately favored during the crystallization process over other possible GRB2 conformations has never been addressed. Taken together, despite crystallographic evidence of a compact GRB2 dimer, our results significantly broaden the spectrum of GRB2 conformational states and reinforce the existence of a novel, full-length, extended form of the GRB2 dimer.

In light of our findings, we derived a model of GRB2 dimer with inter-SH2 domain-swapping informed by the literature that proved to be a better fit for our data. Our model of an SH2-mediated domain-swapped dimer represents an important new component of the continuum of GRB2 structures and augments the existing GRB2 conformational record. What remains to be seen is whether higher-order GRB2 oligomers occur natively or may be induced by better stabilizing the SH2 domain hinge loop into an extended conformation. In a cellular context, our findings raise previously unasked questions about the significance of GRB2 oligomerization and domain-swapping in T cell function. Recognition of antigen by the TCR triggers a cascade of events culminating in the assembly of a large, LAT-nucleated signaling complex referred to as the LAT signalosome. The LAT signalosome is known to be stabilized by multiple cooperative binding events, where one molecule makes multiple binding contacts with other molecules, each of which interacts with one or more other molecules. Each of these interactions occur repeatedly, creating a web of contacts that stabilize the larger signaling cluster. Whether GRB2 oligomerization through domain-swapping exerts any influence on LAT signalosome assembly has never been explored. Three C-terminal LAT phospho-tyrosines (pY171, pY191, and pY226) conform to the GRB2 SH2 motif pYxN and are directly bound by GRB2, whereas the N-terminal pY132 is bound by PLC-γ1. Early studies have shown multi-point binding between GRB2 and phosphorylated LAT, such that a fully-phosphorylated LAT could simultaneously interact with three GRB2 molecules^36^. It has also been shown that least two tyrosines must be phosphorylated in order for phospho-LAT to interact with GRB2^95^. Together, these findings suggest a mechanism of cooperative binding between LAT and GRB2 leading to full engagement of LAT by GRB2 molecules. This raises the interesting possibility that bivalently-phosphorylated LAT may preferentially bind GRB2 dimer over individual GRB2 monomers in order to accelerate full engagement of LAT by GRB2 and precipitate signalosome assembly.

In our studies, we determined that both the phosphorylation of LAT and the clustering of phospho-LAT at the T cell plasma membrane was reduced in cells expressing either of the V123D and V122P/V123P GRB2 mutants, in comparison with WT-expressing cells. The lack of LAT phosphorylation in cells expressing V123D or V122P/V123P GRB2 is interesting. This suggests that the monomer–dimer transitions regulate LAT phosphorylation, but the mechanism underlying this phenomenon is unclear. It is possible that GRB2 conformers either promote LAT phosphorylation or block dephosphorylation of LAT by phosphatases, but further studies are needed identify the mechanism. The most dramatic reduction of phospho-LAT clustering was observed in GRB2-deficient cells, suggesting that the presence of each GRB2 conformer alone was enough to induce at least some LAT clustering. This would also suggest that both forms are needed for optimal LAT clustering, although the details remain unclear. These findings raise several possibilities as to the mechanism underlying the inter-relatedness of GRB2 oligomerization through domain-swapping and T cell signaling. It would be interesting to determine whether interaction between LAT and either GRB2 dimer or monomer variably influences the recruitment of additional GRB2 molecules and potentially other binding partners to the remaining phospho-tyrosine sites; it could be that initial binding of a GRB2 dimer to LAT allows for a speedier engagement of a third GRB2 molecule and PLC-γ1. Another possibility is that co-localization of two GRB2 molecules to LAT itself induces dimerization. Further studies are needed to fully elucidate the sequence and impact of these interactions.

The particular mechanism of SH2-mediated dimerization involving α-helix swapping has been observed in studies of other SH2 domain-bearing signaling proteins that impact T cell signaling^96–98^. Recently, Gads, another member of the GRB2 family of adaptor, was found to bind LAT in a cooperative manner as a dimer. Gads exhibits a similar structure as GRB2, with a central SH2 domain and flanking SH3 domains, except that it contains an additional glutamine- and proline-rich spacer between the SH2 and C-terminal SH3 domains. Gads canonically binds Slp-76 rather than SOS1, but both proteins interact with tyrosine-phosphorylated LAT through their SH2 domains^99–104^. The three LAT phospho-tyrosine sites (pY171, pY191, and pY226) have been shown to bind GRB2 or Gads with comparable affinity, and like GRB2, two of the three sites are required for stable Gads recruitment^31,35^. However, unlike GRB2, where any of the two sites are sufficient, Gads requires two specific sites, 171 and 191, for stable binding. Similar to GRB2, Gads has been found to dimerize in an SH2-dependent manner, where the SH2-mediated dimer was further stabilized by the additional domains found in full-length Gads^96^. Interestingly, the Gads dimer interface was found to be distinct from the phospho-tyrosine-binding pocket such that the binding site remained accessible. Competitive binding experiments revealed preferential binding of the Gads dimer to bivalently-phosphorylated LAT peptide, even in the presence of excess, competing amounts of monovalently-phosphorylated LAT^96^. Furthermore, dimer abrogation through inactivation of the dimerization interface decreased this preferential bivalent binding of Gads to LAT and impaired the ability of Gads to discriminate between monovalently- and bivalently-phosphorylated LAT. Together, these findings imply that like the Gads SH2/SH2 domain-swapped dimer, a GRB2 SH2/SH2 domain-swapped dimer may not only be capable of simultaneously engaging two LAT phospho-tyrosine sites, but that it may prefer to do so. In a cellular setting, inactivation of the dimerization interface disrupted the antigen receptor-induced recruitment of Gads to phospho-LAT and impaired TCR responsiveness, suggesting profound biological consequences for the elimination of SH2-mediated Gads dimerization. Applying these findings to GRB2, it would be interesting to determine whether GRB2, like Gads, shares this ability and preference for engaging bivalently-phosphorylated LAT in the SH2-mediated dimeric form or whether the binding of SH2-mediated GRB2 dimer to phospho-LAT modulates the function of the SH3 domains and/or the availability of the SH3 binding sites. An additional question revolves around whether competition between GRB2 dimer and Gads dimer binding to these shared sites on phospho-LAT influences the overall structure and/or functional output of the LAT signalosome. It is intriguing to consider the possibility that downstream signaling outcomes can be fine-tuned at the level of interactions between phospho-LAT and signaling proteins including GRB2 dimer or monomer.

Our studies open new lines of investigation into the oligomeric states and consequent functional integrity of many other SH2 domain-swapped signaling molecules^97,98,105^. SH2-mediated dimerization may represent a new mechanism through which signal transduction pathways are formed, controlled, and sensitized to discriminated between weak signals and authentic environmental triggers. Further studies will be needed to delineate the full biological function of SH2 domain-swapped GRB2 and the details underlying the mechanisms by which GRB2 oligomerization modulates T cell processes. It will also be interesting to determine whether SH2 domain-swapping may serve as a branchpoint through which larger GRB2 networks may form. Future directions for this work will revolve around complementing these findings with methods that provide increased resolution, such as crystallography, cryoEM, and/or NMR. Additionally, hydrogen–deuterium exchange mass spectrometry (HDX) would allow for empirical determination of flexible regions in the SH2 domain-swapped dimer.

## Methods

### Cell culture and stimulation

HUT 78 T lymphocytes were cultured at 37 °C and 5% CO_2_ in complete RPMI media (RPMI 1640 supplemented with 10% FBS, 100 U/mL penicillin, 100 μg/mL streptomycin, and 2 mM L-glutamine) (Gibco). Cells were maintained at concentration of 0.1–1 × 10^6^ cells/mL. HEK 293 T cells were cultured under the same conditions in complete DMEM media (DMEM supplemented with 10% FBS, 100 U/mL penicillin, 100 μg/mL streptomycin, and 2 mM L-glutamine, and 1X MEM NEAA) (Gibco). Cells were maintained at < 80% confluency. For experiments, cells were stimulated at a density of 0.5 × 10^6^ cells/mL with 5 μg/mL anti-CD3 antibody (BioLegend) immobilized in 6-well or 12-well tissue culture plates for 18–24 h prior to analysis. Unless otherwise stated, cells were lysed in hot 1X Laemmli buffer (BioRad), heated at 95 °C for 5 min, sonicated to reduce viscosity, and then stored at − 20 °C prior to electrophoresis or analyzed immediately.

### Cloning

For mammalian expression, HUT 78 T lymphocytes were transduced with pLK4 lentiviruses containing GRB2 shRNAs alone or with sequences encoding add-back wild-type or mutant GRB2 as previously described^106^. Six silent mutations were introduced to the add-back GRB2 sequences to generate GRB2 messenger resistant to shRNA-mediated degradation. GRB2 derivatives bearing the mutations discussed were constructed by site-directed mutagenesis. The V123D putative monomer mutation was introduced by PCR using the following primers: 5′-GAAGTACTTCCTCTGGGTGGATAAGTTCAATTC-3′ and 5′-AGCTCATTCAAAGAATTGAACTTATCCACCCAGAG-3′. For the V122P/V123P putative dimer mutant, the primers were the following: 5′-CGGGAAGTACTTCCTCTGGCCGCCGAAGTTCAATT-3′ and 5′-GCTCATTCAAAGAATTGAACTTCGGCGGCCAGAGGAAG-3′. The N188D mutation was introduced by overlap extension PCR using the following primer pairs: 5′-CTTCAAGGTGCTCCGAGATGGAG-3′ and 5′-GGGTCTGAGTCATCCATGACATGGATAAAATCTC-3′; 5′-GTCATGGATGACTCAGACCCCAACTGGTG-3′ and 5′-GGGCGACCGGACTCTAGAG-3′. The N214D mutation was introduced using the following primers: 5′-GGACATAGAACAGGTGCCACAGCAG-3′ and 5′-AGTCGCGGCCGCTTAGACGTTCCGGTCCACGGGGGTGAC-3′. Primers were purchased from Integrated DNA Technologies (Coralville, IA). Expression of shRNA was under control of the U6 promoter, whereas add-back GRB2 expression was under control of the EF-1α promoter. Transduced cells were kept in selection with 1 μg/mL puromycin (Santa Cruz). For bacterial expression, the sequence encoding full-length, wild-type human GRB2 was cloned into the pET-28a(+) vector (Novagen, cat. no. 69864) and *E. coli* BL21 (DE3) cells transformed with the vectors as previously described^36^. The same primers as listed above were used to generated Histidine-tagged variants of GRB2 for recombinant expression.

### Recombinant protein expression, immobilized metal ion affinity chromatography (IMAC), and size exclusion chromatography (SEC)

Bacterial cells transformed with human His-tagged GRB2 were grown on an LB plate supplemented with 50 μg/mL kanamycin that was inverted and incubated at 37 °C overnight. A starter culture of 5 mL of Superbroth (SB) medium with antibiotic was inoculated with a single clone and incubated at 37 °C with shaking at 250 rpm for 6–8 h, or until cloudy. The starter culture was then inoculated into 1 L of medium and cells grown at 37 °C with shaking for 3–4 h to an OD_600_ of 0.6–0.8. Cultures were then cooled to 20 °C, induced with 500 uM IPTG, and allowed to grow for 18–24 h. Cells were then pelleted and frozen at − 80 °C prior to protein isolation. Pellets were thawed in a hot waterbath for 2–5 min and resuspended in cold lysis buffer (50 mM sodium phosphate, pH 8.0, 250 mM NaCl, 2 mM bME) supplemented with EDTA-free protease inhibitor cocktail (Roche) at 60 mL per 1 L of culture. Resuspensions were subjected to 4 rounds of sonication (30 s on/30 s off) and centrifuged at 18,000 rpm at 4 °C for 1 h. Supernatants were clarified with 0.45 um filters and applied to a 5 mL Ni–NTA agarose column. All subsequent steps were performed at room temperature. Protein was eluted over a linear gradient of 25 mM to 250 mM imidazole. Fractions containing GRB2 were pooled and concentrated using Amicon Ultra-15 centrifugal filter units (MWCO 10 kDa) (Millipore). Particular care is required for sample preparation from this point on, given the increasingly unfavorable conditions (e.g., dialysis for the removal of L-arginine following SEC) and thus, increasing potential for precipitation, as has been reported^64,70^. The protein was further purified by SEC and the concentrate injected onto a 120 mL Superdex 75 10/300 preparative-grade gel filtration column (GE) equilibrated with 20 mM Tris pH 8.0, 150 mM NaCl, 100 mM L-arginine, and 1 mM DTT on a Bio-Rad BioLogic DuoFlow chromatography system. The apparent molecular weight of the protein was approximated using a calibration curve generated with known standards. Fractions containing the protein were again pooled and concentrated using Amicon filters in preparation for dialysis. The protein was dialyzed at room temperature at 500× the sample volume into buffers of gradually decreasing L-arginine concentration: 25 mM, 10 mM, and 0 mM. Protein aliquots in 30% glycerol were flash-frozen and stored at − 80 °C, or used immediately in experiments. Aliquots were reserved at various points throughout the isolation process for SDS–PAGE to determine protein purity and integrity. Samples were diluted with hot 4× Laemmli buffer, heated at 95 °C for 5 min, and then stored at − 20 °C prior to electrophoresis or analyzed immediately. Samples were then loaded onto pre-cast 4–20% Mini-PROTEAN TGX Stain-free polyacrylamide gels (Bio-Rad) and run at 120 V for up to 1 h prior to visualization of resolved bands using the Bio-Rad Gel Doc EZ Imager.

### Calcium phosphate transfection, lentivirus production, and mammalian cell transduction

To generate pseudo-typed lentiviral particles, shRNA-encoding pLK4 vectors were transfected into 293 T cells along with pCL-Eco and Pax2 packaging vectors and VSV-G envelope vector. At 18–24 h prior to transfection, 7.5 × 10^6^ cells were seeded into one 15 cm tissue culture dish in cDMEM and incubated overnight. At 1–2 h prior to transfection, the media was replaced with fresh cDMEM. Lentiviral DNA was introduced into 293 T cells using the calcium phosphate transfection method. Per transfection, plasmids were combined at a ratio of 6:4:3 (30 ug lentiviral plasmid containing the desired construct, 20 ug packaging vectors, and 15 ug envelope vector) with CaCl_2_ at a final concentration of 125 mM and HEPES-buffered saline at a final concentration of 50 mM HEPES pH 7.05, 140 mM NaCl, and 1.5 mM Na_2_HPO_4_). DNA was allowed to precipitate for 30 min at room temperature, then added drop-wise to 293 T cells. After 12–18 h, the transfection media was replaced with fresh cDMEM media combined with unsupplemented DMEM at a ratio of 20/80 for a final FBS concentration of 2%. Viral supernatant was harvested at 24 h intervals over the following 3 days, then spun down and filtered through 0.45-um Durapore Millex (Millipore) filters. Virus was diluted in a solution of 10% (w/v) PEG-8000 and 300 mM NaCl, rotated overnight at 4 °C, and spun down at 3000 g for 1 h at 4 °C. The viral pellet was held overnight at 4 °C in 1× PBS at 1/100th the original volume of the viral supernatant and spun down to pellet serum protein and other particulates. Concentrated virus was stored at -80 °C or used immediately to transduction. HUT 78 T lymphocytes at 0.5 × 10^6^ cells/mL were allowed to grow for 2–3 days in the presence of concentrated virus and hexadimethrine bromide (Polybrene) transfection reagent (Sigma Aldrich) at a concentration of 8 μg/mL before removal of the virus and initiation of puromycin selection. Cells were allowed to expand in the presence of increasing puromycin concentration, from 0.25 μg/mL to a final selection concentration of 1 μg/mL. Whole cell lysates were probed for loss of GRB2 expression and expression of GRB2 mutants after 2–3 passages in puromycin.

### Cytokine detection by ELISA

Cells were washed in complete RPMI 1640, and then resuspended at 2–5 × 10^5^ cells/mL. Cells were stimulated by adding 0.5 mL of cell suspension to 24-well plates coated with anti-CD3 for 24 h. IL2 protein concentrations in culture supernatants were measured using standard TMB ELISA utilizing a spectrophotometric plate reader with a reading absorbance at 450 nm.

### Western blot and antibodies

Following SDS-PAGE, resolved proteins were transferred onto a PVDF membrane (Millipore) and then blocked for 1 h at room temperature in a 1:1 solution of SEA Block buffer (Thermo Scientific) in 1X PBS. Membranes were incubated for 1 h at room temperature or overnight at 4 °C with primary antibodies followed by two washes in 0.05% Tween-20 in 1X PBS. Secondary anti-mouse or anti-rabbit DyLight 680- or 800-conjugated antibodies were applied for 1 h at room temperature followed by 2 washes. Blots were then visualized using the Licor Odyssey Infrared detector. Densitometric analysis of protein bands was performed using Odyssey’s v3.0 software and normalized to GAPDH. The followed primary antibodies were used: GRB2 (BD Pharmingen or Cell Signaling Technology); LAT pY226 (clone J96-1238.58.93, BD Pharmingen); and GAPDH (Meridian Bioscience).

### Size exclusion chromatography coupled with multi-angle light scattering and small-angle X-ray scattering (SEC–MALS–SAXS)

SEC–MALS–SAXS data sets were collected using the 18-ID-D BioCAT Beamline at the Advanced Proton Source (APS) at Argonne National Laboratory (Chicago, IL). Samples were centrifuged for 5 min at 13,000 rpm to remove any potential aggregates prior to column loading. Samples containing 4–9 mg/mL of GRB2 WT or mutants in 250 μL were injected onto a 24 mL Superdex 75 Increase 10/300 analytical-grade column (GE) equilibrated with 20 mM Tris pH 8.0, 150 mM NaCl, and 1 mM DTT at a flow rate of 0.5 mL/minute on an Agilent 1300 chromatography system. Column eluant was analyzed in line by the UV absorbance detector of the Agilent 1300 chromatography system, then subsequently directed into the DAWN Heleos-II light scattering (LS) and OptiLab T-rEX refractive index detectors in series. Finally, the elution trajectory directed samples into a 1.0 mm ID quartz capillary SAXS sample cell. Scattering data were collected every 1 s using a 0.5 s exposure and detected with a Pilatus 3 1 M pixel detector (DECTRIS) with a 12 keV (1.033 Å wavelength) X-ray beam covering a q-range of 0.0045 < q < 0.35 Å − 1 (q = 4π/λsinθ, where λ is the wavelength and 2θ is the scattering angle). Accurate protein molecular weights from MALS data were determined using the ASTRA software (Wyatt Technology).

### Small-angle X-ray scattering (SAXS) data processing and modeling

SAXS data reduction, buffer subtraction, and further analysis were performed using BioXTAS RAW version 2.1.1^107^. An average of 30 frames prior to an eluted peak was used for buffer subtraction. Protein peaks were also run through evolving factor analysis (EFA) to deconvolute peaks into the individual scattering components where applicable. The forward scattering intensity I(0) and radius of gyration (*R*_*g*_) were calculated from the Guinier fit. The normalized Kratky plot, pair distance distribution plot, or *P(r)*, and Porod volume (*V*_*P*_) were calculated using the program GNOM embedded in the BioXTAS RAW software^86^. The calculation of theoretical scattering curves for the crystal structure PDB 1GRI was performed using the program CRYSOL, part of the ATSAS software package (version 3.1.0)^86,87^. The initial all-atom model of the full-length SH2/SH2 domain-swapped dimer was generated with PyMOL version 2.5.2 using PDB structures 1GRI (full-length GRB2 dimer) and 6ICH (SH2 domain-only dimer)^60,71,108^. Each chain of the 1GRI dimer was superimposed over one of the SH2 domains comprising the 6ICH dimer. Intermodular linkers were built, N-terminal His-tags added, and missing amino acids inserted using YASARA^109^. Reconstruction of the electron density was calculated from SAXS data using the program DENSS version 1.6 embedded in the BioXTAS RAW software^89^. The final representative model was colorized and annotated using PyMOL and Chimera version 1.16^110^.

## Supplementary Information

Supplementary Information.

## Data Availability

SAXS datasets, experiment details, and atomic model and fits have been deposited in the Small Angle Scattering Biological Data Bank (SASBDB)^112,113^. The data are available under the following accession codes at the following links: GRB2 WT monomer (SASDP28; https://www.sasbdb.org/data/SASDP28/034i8jy0aw), GRB2 WT dimer (SASDP38; https://www.sasbdb.org/data/SASDP38/nzgrpqf3qh), GRB2 N188D/N214D monomer (SASDP48; https://www.sasbdb.org/data/SASDP48/yx3pkg8xnx), GRB2 N188D/N214D dimer (SASDP58; https://www.sasbdb.org/data/SASDP58/laf3oekd7k), GRB2 V123D monomer (SASDP68; https://www.sasbdb.org/data/SASDP68/wo1wxpdnm6), and GRB2 V122P/V123P dimer (SASDP78; https://www.sasbdb.org/data/SASDP78/mkqxjzp801).
