# Supplementary material for: GRB2 dimerization mediated by SH2 domain-swapping is critical for T cell signaling and cytokine production
Source: Sci Rep. 2023 Mar 2;13:3505. doi: 10.1038/s41598-023-30562-7 (PMC9981690; doi:10.1038/s41598-023-30562-7)
Supplement: Supplementary file 1 — Supplementary Information. [file 41598_2023_30562_MOESM1_ESM.pdf]

## Figure Legends

*Supplemental Figure 1.* SDS-PAGE analysis following capture by IMAC of full-length, His-tagged GRB2 A) WT, B) N188D/N214D, C) V123D, and D) V122P/V123P. Loading order was as follows: bulk lysate (lane 1); post-capture flow-through (lane 2); eluate (lanes 3-5 and 7-10 or 11); molecular weight ladder (lane 6). Red starts denote target protein in lysate, absent thereafter in flow-through. Analyses representative of at least 3 independent experiments.

*Supplemental Figure 2.* SDS PAGE analysis of fractions spanning chromatographic peaks are shown for A) GRB2 WT, B) N188D/N214D, C) V123D putative monomer, and D) V122P/V123P putative dimer.

*Supplemental Figure 3.* SEC-MALS elutions of GRB2 dimers in full, with light scattering and refractive index plotted against elution time for A) WT dimer, B) N188D/N214D dimer, and C) V122P/V123P dimer. Dashed lines denote the molecular weights of GRB2 monomer (~28 kDa) and dimer (~56 kDa).

*Supplemental Figure 4.* Guinier analyses of the low-q regions, along with $R^2$ are shown for GRB2 A) WT monomer, B) WT dimer, C) N188D/N214D monomer, D) N188D/N214D dimer, E) V123D putative monomer, and F) V122P/V123P putative dimer.

*Supplemental Figure 5.* Western immunoblot of whole-cell lysates (WCL) of transfected HuT78 T cells probing for GRB2 and GAPDH (control). Loading order was as follows: molecular weight ladder (lane 1); WCL of GRB2 knockdown (lane 2); WCL of GRB2 WT addback (lane 3); WCL of GRB2 V123D addback (lane 4); WCL of GRB2 V123N addback (lane 5); and WCL of GRB2 V122P/V123P addback (lane 6). Note: GRB2 V123N mutant was not used in these studies.

*Supplemental Figure 6.* Western immunoblot of whole-cell lysates (WCL) of transfected HuT78 T cells probing for phospho-LAT (pY226) and GAPDH (control).

*Supplementary Table 1.* Mammalian GRB2 structures available in the PDB.

*Supplemental Table 2.* SEC-MALS-SAXS experimental parameters.

# Supplemental Figure 1

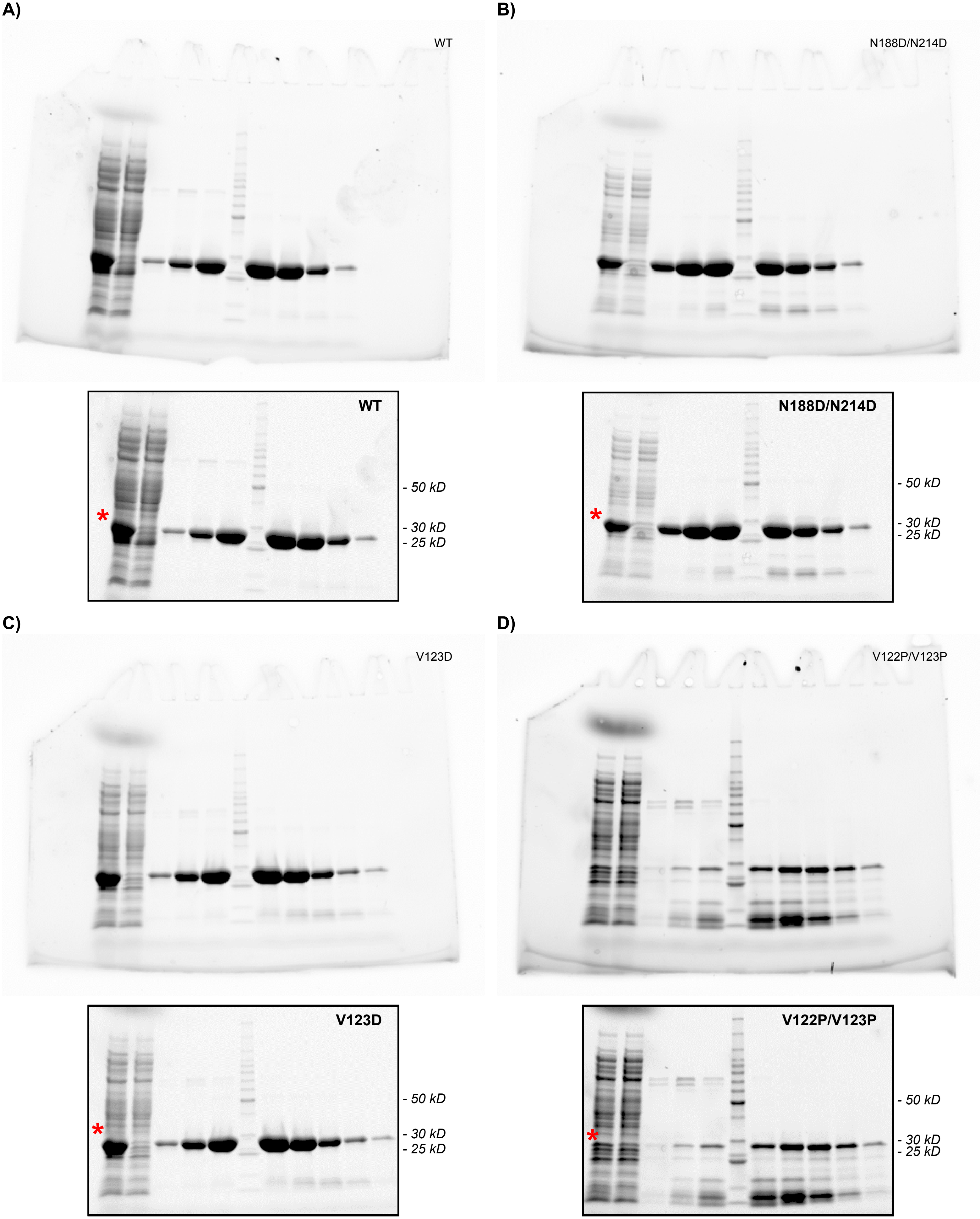

# Supplemental Figure 2

A)

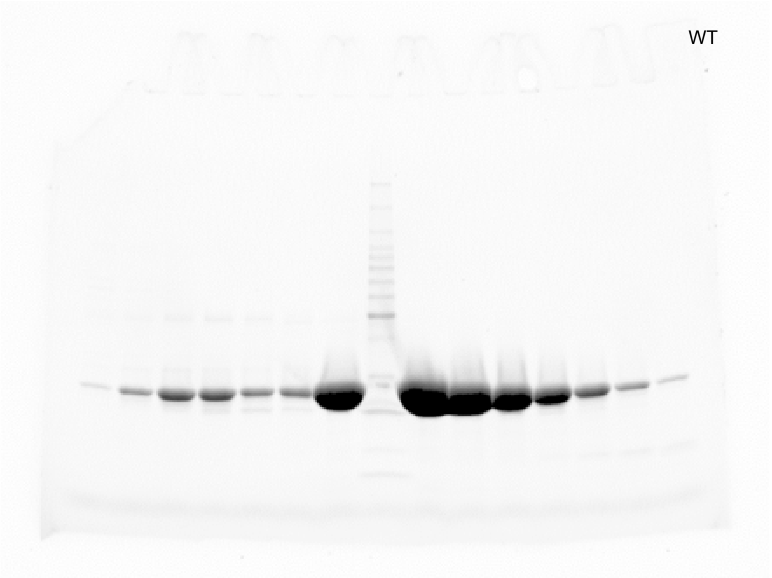


B)

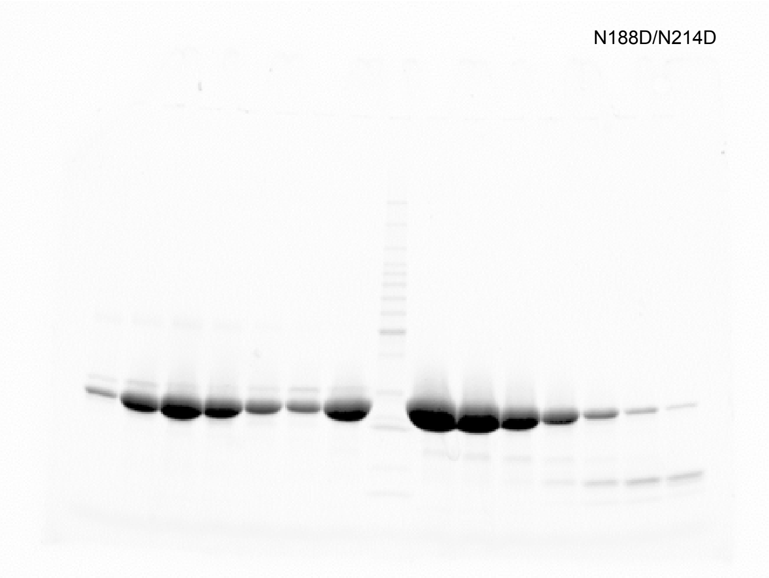


C)

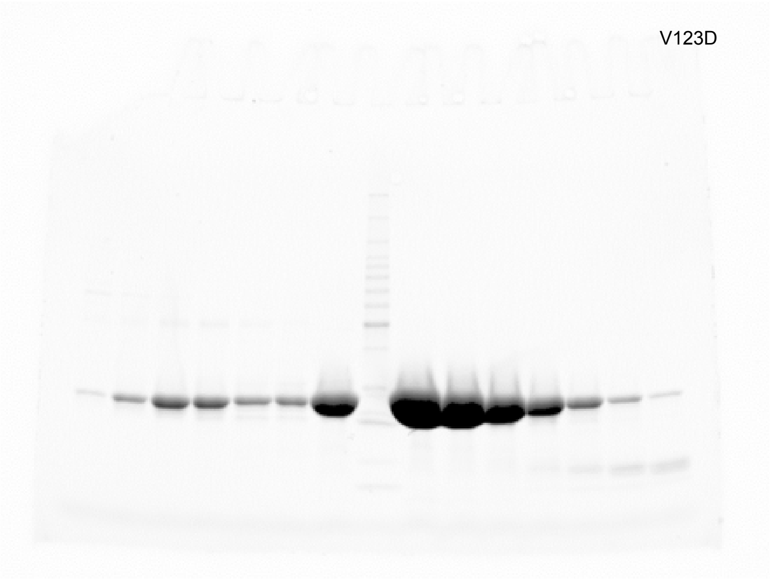


D)

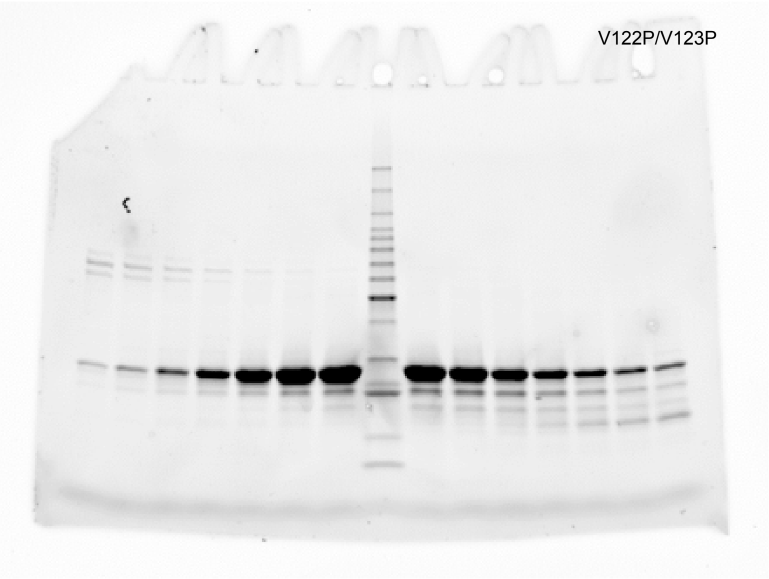

# Supplemental Figure 3

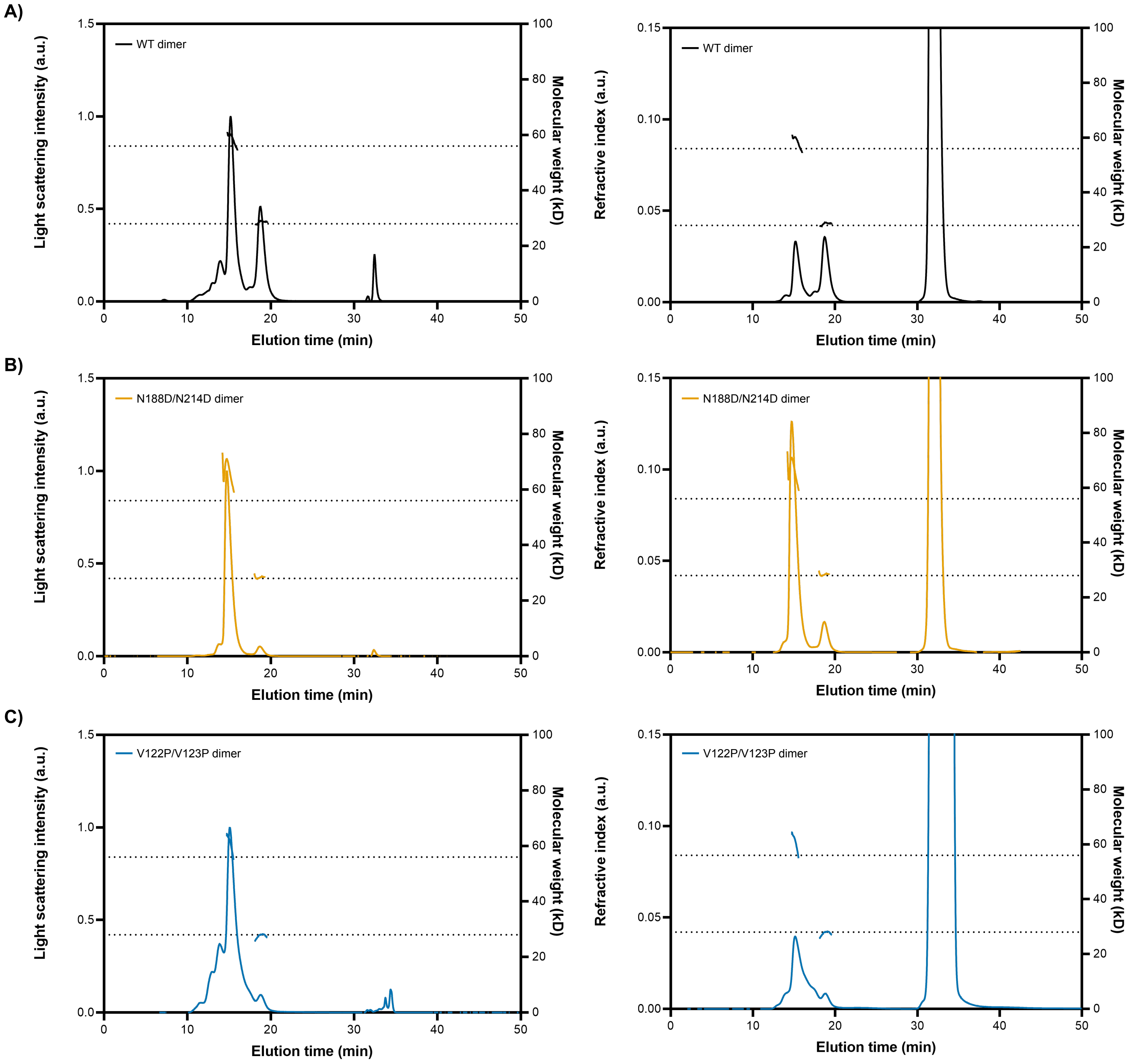

# Supplemental Figure 4

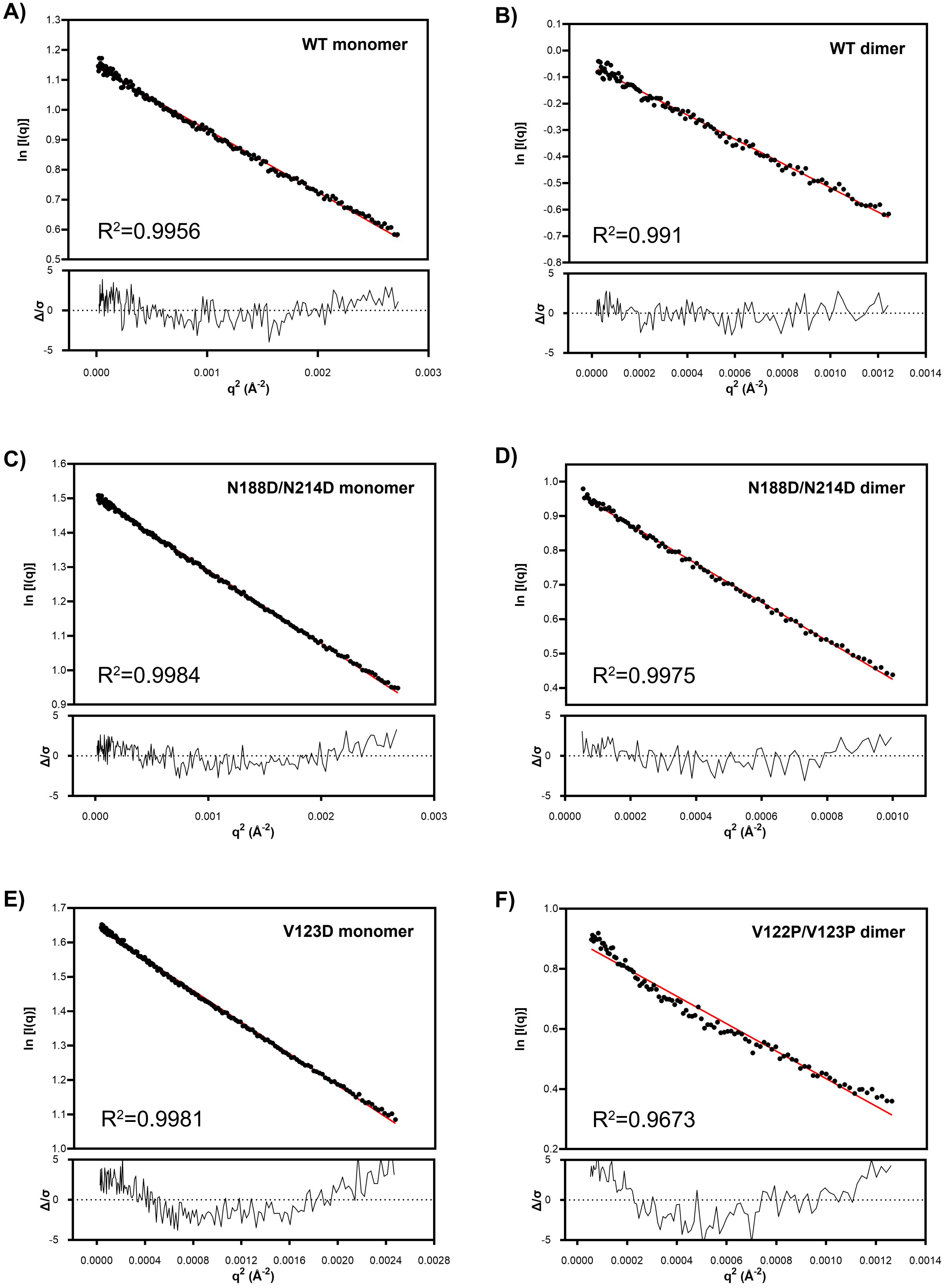

# Supplemental Figure 5

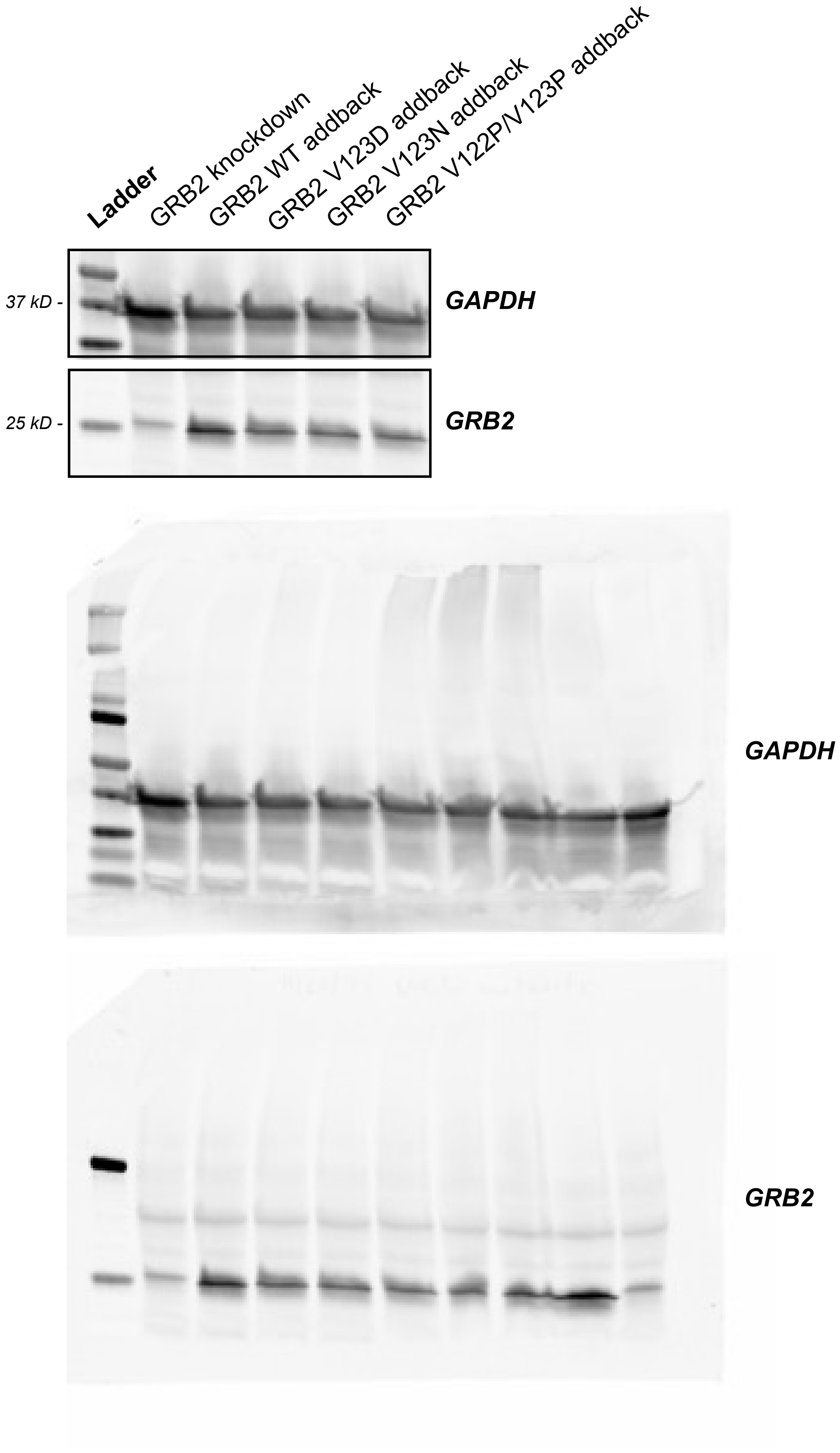

# Supplemental Figure 6

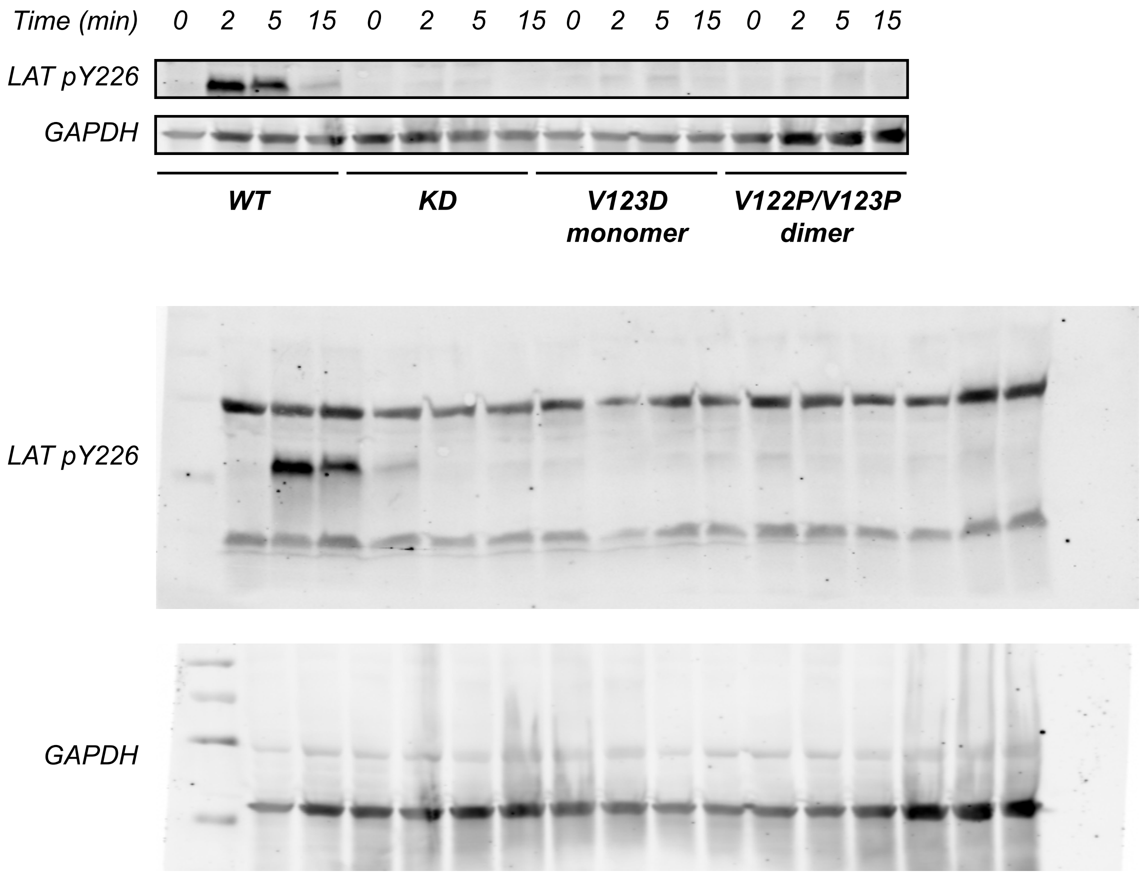

# Supplemental Table 1

| *PDB ID* | *Resolution (Å)* | *Number of entities* | *Molecular weight (kD)* | *Domain(s) represented* | *Residues represented* | *Ligand* | *Reference (PMID)* |
|---|---|---|---|---|---|---|---|
| 1BM2 | 2.1 | 2 | 14.42 | SH2 | 49-163 | Yes | 10090780 |
| 1BMB | 1.8 | 2 | 15.57 | SH2 | 49-168 | Yes | 10090780 |
| 1CJ1 | 3 | 1 | 142.16 | SH2 | 57-152 | Yes | 10395476 |
| 1FYR* | 2.4 | 2 | 55.52 | SH2 | 50-161 | Yes | 11063574 |
| 1GRI* | 3.1 | 1 | 50.48 | SH2 | 1-217 | No | 7716522 |
| 1JYQ | 2 | 2 | 23.67 | SH2 | 60-151 | Yes | 11827484 |
| 1JYR | 1.55 | 2 | 12.13 | SH2 | 60-151 | Yes | 11827484 |
| 1JYU | 2.75 | 1 | 11.07 | SH2 | 60-151 | No | 11827484 |
| 1TZE | 2.1 | 2 | 12.39 | SH2 | 55-152 | Yes | 8673601 |
| 1ZFP | 1.8 | 2 | 12.37 | SH2 | 56-153 | Yes | 9642078 |
| 2AOA | 1.99 | 1 | 25.74 | SH2 | 55-153 | Yes | 16165154 |
| 2AOB | 1.8 | 1 | 55.36 | SH2 | 55-153 | Yes | 16165154 |
| 2H46* | 1.9 | 1 | 13.78 | SH2 | 53-162 | No | 17466257 |
| 2H5K | 3.25 | 2 | 28.01 | SH2 | 53-162 | Yes | 17466257 |
| 2HUW | 1.9 | 1 | 28.52 | SH2 | 53-162 | Yes | 17001728 |
| 3C7I | 1.7 | 1 | 14.26 | SH2 | 53-162 | Yes | 17001728 |
| 3IMD | 2 | 1 | 28.8 | SH2 | 53-163 | Yes | 19886660 |
| 3IMJ | 2.02 | 1 | 28.6 | SH2 | 53-163 | Yes | 19886660 |
| 3IN7 | 2 | 1 | 28.63 | SH2 | 53-163 | Yes | 19886660 |
| 3IN8 | 1.7 | 1 | 14.35 | SH2 | 53-163 | Yes | 19886660 |
| 3KFJ | 2.02 | 1 | 14.41 | SH2 | 53-163 | Yes | 19886660 |
| 3MXC | 2 | 2 | 12.89 | SH2 | 55-152 | Yes | 22001015 |
| 3MXY | 2.3 | 2 | 12.86 | SH2 | 55-152 | Yes | 22001015 |
| 3N7Y | 2.02 | 2 | 36.44 | SH2 | 55-152 | Yes | 21116482 |
| 3N84 | 2 | 2 | 83.89 | SH2 | 53-163 | Yes | 21116482 |
| 3N8M | 2 | 2 | 14.63 | SH2 | 53-163 | Yes | 21116482 |
| 3OV1 | 1.6 | 2 | 14.51 | SH2 | 53-163 | Yes | 22007755 |
| 3OVE | 1.82 | 2 | 14.2 | SH2 | 53-163 | Yes | 22007755 |
| 3S8L | 1.71 | 2 | 14.38 | SH2 | 53-163 | Yes | 22007755 |
| 3S8N | 1.71 | 2 | 14.62 | SH2 | 53-163 | Yes | 22007755 |
| 3S8O | 1.85 | 2 | 14.34 | SH2 | 53-163 | Yes | 22007755 |
| 3WA4 | 1.35 | 2 | 12.58 | SH2 | 60-152 | Yes | 24098653 |
| 4P9V | 1.64 | 2 | 14.45 | SH2 | 53-163 | Yes | 24856058 |
| 4P9Z | 1.8 | 2 | 14.69 | SH2 | 53-163 | Yes | 24856058 |
| 5CDW | 2.602 | 2 | 197.82 | SH2 | 54-153 | Yes | 26645482 |
| 6ICG | 1.15 | 1 | 22.57 | SH2 | 60-152 | No | 30923665 |
| 6ICH* | 2 | 1 | 11 | SH2 | 60-152 | No | 30923665 |
| 6WM1 | 1.8 | 2 | 29.06 | SH2 | 53-163 | Yes | 32916312 |
| 6WO2 | 2 | 2 | 28.92 | SH2 | 53-163 | Yes | 32916312 |
| 1GCQ | 1.68 | 2 | 22.15 | C-SH3 | 159-217 | Yes | 11406576 |
| 2VVK | 1.6 | 1 | 6.46 | C-SH3 | 161-214 | No | 19523899 |
| 2VWF | 1.58 | 2 | 8.39 | C-SH3 | 159-214 | No | 19523899 |
| 2W0Z | 1.7 | 2 | 7.57 | C-SH3 | 159-214 | No | 19523899 |
| 6SDF | 2.5 | 1 | 14.7 | N-SH3 | 2-59 | No | 32510467 |

*Domain-swapped dimer

# Supplemental Table 2

| | |
|---|---|
| **A) Sample details** | |
| SEC Column | Superdex 75 increase 10/300 |
| Loaded concentration (mg/ml) | 8.28 (WT monomer), 4.46 (WT dimer), 8.37 (N188D/N214D monomer), 2.84 (N188D/N214D dimer), 8.68 (V123D monomer), 4.00 (V122P/V123P dimer) |
| Injection volume (ul) | 250 |
| Flow rate (ml/min) | 0.5 |
| Solvent (solvent blanks taken from SEC flow-through prior to elution of protein) | 20 mM Tris pH 8.0. 150 mM NaCl, 1 mM DTT |
| **B) SAXS data collection parameters** | |
| Instrument | BioCAT facility at the Advanced Photon Source beamline 18ID with Pilatus3 1M (Dectris) detector |
| Wavelength (Å) | 1.033 |
| Beam size ($um^2$) | 150 (h) x 80 (v) |
| Camera length (m) | 3.5 |
| $q$ measurement range ($Å^{-1}$) | 0.0045-0.35 |
| Absolute scaling method | N/A |
| Basis for normalization to constant counts | To incident intensity, by ion chamber counter |
| Monitoring for radiation damage | Frame-by-frame comparison of data |
| Exposure time | 0.5 sec exposure time with 1 sec total exposure period of entire SEC elution |
| Sample configuration | SEC-MALS-DLS-RI-SAXS. Size separation used a Superdex 75 increase 10/300 GL column and an Infinity II HPLC (Agilent Technologies). Flow was in line with the UV-MALS-DLS-RI instruments and SAXS after the column. UV data was measured in the Agilent, and MALS-DLS-RI data by DAWN HELEOS-II (17 MALS + 1 DLS channels) and Optilab T-rEX (RI) instruments (Wyatt). SAXS data was measured with sheath-flow cell in a 1.5 mm ID 1.52 mm OD quartz capillary, effective path length 0.49 mm. |
| Sample temperature (°C) | 22 |
| **C) Software employed for SAXS data reduction, analysis, and interpretation** | |
| SAXS data reduction | Radial averaging, background subtraction, frame comparison, averaging, and subtraction were made using BioXTAS RAW 2.0.2. |
| Basic analysis: Guinier, MW, Normalized Kratky, P(r) | Guinier plot and molecular weights were calculated using BioXTAS RAW 2.1.1, P(r) function using GNOM from ATSAS 3.0. |
| MALS-DLS-RI analysis | Astra 7.1.3 (Wyatt) |
